# Switching from ritonavir to cobicistat in HIV patients with renal impairment who are virologically suppressed on a protease inhibitor

**DOI:** 10.7448/IAS.17.4.19824

**Published:** 2014-11-02

**Authors:** Martin Fisher, Cheryl McDonald, Graeme Moyle, Claudia Martorell, Moti Ramgopal, Francois Laplante, Joanne Curley, Hiba Graham, Cecilia Tran-Muchowski, Yapei Liu, Martin Rhee, Javier Szwarcberg

**Affiliations:** 1 Department of HIV and Genitourinary Medicine, Brighton and Sussex Medical School, Foster City, CA, USA; 2 Department of Medicine, Tarrant County Infectious Disease Associates, Fort Worth, TX, USA; 3 Department of HIV Medicine, Chelsea and Westminster Hospital, London, UK; 4 Department of Medicine, The Research Institute, Springfield, IL, USA; 5 Department of Medicine, Midway Immunology and Research Center, Fort Pierce, FL, USA; 6 Department of Medicine, Clinique Medicale du Quartier Latin, Montreal, WI, USA; 7 Department of Project Management, Gilead Sciences, Foster City, CA, USA; 8 Department of Clinical Research, Gilead Sciences, Foster City, CA, USA; 9 Department of Clinical Operations, Gilead Sciences, Foster City, CA, USA; 10 Department of Biometrics, Gilead Sciences, Foster City, CA, USA

## Abstract

**Introduction:**

Cobicistat (COBI) is a pharmacoenhancer and one of the components of ECF/TDF (elvitegravir/cobicistat/emtricitabine/tenofovir DF), which is approved in treatment-naïve HIV patients with creatinine clearance (CrCl) ≥70 mL/min. Study 118 assessed the renal safety of COBI-containing regimens in HIV patients with mild to moderate renal impairment.

**Material and Methods:**

Phase 3, open label study in HIV-1-infected patients with CrCl 50–89 mL/min who are virologically suppressed on a stable regimen containing ritonavir (RTV)-boosted atazanavir (ATV) or darunavir (DRV). Patients switched RTV to COBI, while keeping the rest of their regimen unchanged. We present the 96-week (Wk) data.

**Results:**

Seventy-three patients were enrolled. Mean age was 54 years; male 82%; white 77%; hypertension 38%; diabetes 18%; baseline proteinuria (≥trace) 51%; median CrCl 71 mL/min (range: 42–98). At Wk 96, 89% maintained virologic suppression (95% CI 77.4–95.8%). No emergent resistance developed. Reductions in CrCl (median [IQR]) were observed as early as Wk 2, after which they were nonprogressive through Wk 96 (Wk 48: −3.8 [−9–0.8]; Wk 96: −5.0 [−13.0–0.1]). Changes in CrCl by baseline CrCl (<70 vs ≥70) at Wk 96 were: −3.1 [−5.1–0.5] vs −7.6 [−15.2 to −3.6], respectively. Cystatin C-based eGFR remained stable through Wk 96 (median [IQR]: −2.8 [−7.4–8.9 mL/min/1.73 m^2^). Actual GFR assessment using CL_iohexol_ (n=14) was unaffected over 24 Wks (median at baseline: 82.5, median changes from baseline at Wks 2, 4, and 24: 1.6, 7.0, −4.1 mL/min, respectively). Three renal discontinuations occurred (two worsening CrCl and one proteinuria/hematuria); none had proximal renal tubulopathy [PRT]. No patient had laboratory evidence of PRT (>1 confirmed renal laboratory abnormalities [increase in serum Cr≥0.4 mg/dL, ≥2-grade increase in proteinuria,≥1-grade increase in normoglycemic glycosuria or hypophosphatemia]).

**Conclusions:**

In HIV-infected patients with CrCl 50–89 mL/min, on ATV- or DRV-based regimen switching to COBI from RTV, demonstrated that COBI was well tolerated with no cases of PRT through 96 Wks. The renal safety profile of COBI in patients with mild to moderate renal impairment was consistent with the long-term data in patients without renal impairment (CrCl≥70 mL/min) from the phase 3 studies of COBI-containing regimens.

